# Editorial: New trends in single photon emission computed tomography (SPECT)

**DOI:** 10.3389/fmed.2023.1349877

**Published:** 2023-12-20

**Authors:** Roberto Massari, Greta S. P. Mok

**Affiliations:** ^1^Institute of Biochemistry and Cell Biology, National Research Council of Italy (CNR), Rome, Italy; ^2^Biomedical Imaging Laboratory (BIG), Department of Electrical and Computer Engineering, Faculty of Science and Technology, University of Macau, Taipa, Macao SAR, China; ^3^Center for Cognitive and Brain Sciences, Institute of Collaborative Innovation, University of Macau, Taipa, Macao SAR, China; ^4^Ministry of Education Frontiers Science Center for Precision Oncology, Faculty of Health Science, University of Macau, Taipa, Macao SAR, China

**Keywords:** single photon emission computed tomography, QSPECT, fast SPECT, artificial intelligence-AI, SPECT/CT

Single photon emission computed tomography (SPECT) is a nuclear imaging technique that provides three-dimensional information about the functional and molecular processes within the patient's body. In SPECT, one or more gamma cameras are used to detect mostly gamma radiation from the administered radioactive tracer. The detectors rotate around the patient, acquiring projections from multiple angles for then three-dimensional reconstruction of the radioactive tracer distribution.

This technique is deeply rooted in the development of nuclear medicine imaging. The very first demonstration of SPECT was carried out by Kuhl and Edwards (1), although it was not until the late 1970s that the first commercial scanner based on rotating gamma camera was developed at Searle Radiographics (2). Nowadays, SPECT imaging is an integrated diagnostic routine in nuclear medicine, where the gamma camera is the workhorse due to its flexibility to perform both planar and tomographic imaging.

However, the development of SPECT imaging has been surpassed in some ways by positron emission tomography (PET) in recent years, which has gained considerable attention in the field of nuclear medicine (3). PET has higher sensitivity, provides high image quality with better spatial resolution as well as more quantitative capability than SPECT. These factors may make PET a more attractive option for certain applications. One factor that has indirectly influenced the rise of PET in past years is the shortage of isotopes in nuclear medicine, a significant issue that has affected the availability of essential medical procedures (4). In recent years, the production of isotopes for nuclear medicine has suffered a series of unplanned interruptions associated with the aging of the medical nuclear reactors, despite all efforts to maintain these infrastructures. These interruptions caused severe shortages of ^99^Mo, used in ^99m^Tc generators, resulting in the cancellation of many ^99m^Tc-based SPECT studies. On the other hand, PET is more reliable as most PET tracers, e.g., ^18^F, are produced by cyclotron and are less prone to shortages. However, purchasing and maintaining a cyclotron is expensive and requires substantial engineering support, which may not be feasible for most clinical institutions.

Despite the above, SPECT are almost ubiquitous in hospitals and clinics and are more affordable than PET. The SPECT technique uses gamma-emitting radioisotopes, which have longer half-lives than the short-lived β^+^ isotopes used in PET. This allows for a longer imaging time to capture the patients' pharmacokinetics. In addition, SPECT is able to perform multi-tracer studies, which can provide more comprehensive information on the biological processes being examined.

Moreover, SPECT can be effectively combined with computed tomography (CT), which offers morphological details and solution of attenuation correction, as an integrated dual-modality scanner known as SPECT/CT (5). This leads to enhanced sensitivity (improved detection of the disease) and specificity (reduction of false positives due to normal uptake of the tracer). In this regard, the first commercial SPECT/CT system was the Hawkeye scanner, introduced in 1999 by GE Healthcare (then known as GE Medical Systems) (6).

Although SPECT is well-established and widely validated, it is still a promising technique that is evolving with ongoing technological advances and research. This Research Topic encompasses several contributions highlighting the most recent progress in SPECT, providing a broad overview of advances in instrumentation, use of artificial intelligence (AI) techniques, quantitative imaging, and exploration of new applications.

In recent years, SPECT detectors have benefited from many technological advances. For instance, new scintillator materials with better properties have been developed and, thanks to advances in solid-state devices, new light transducers such as silicon photomultipliers (SiPM) or new semiconductor-based detectors such as cadmium zinc telluride (CZT) have become available (7). More specifically, these enhanced components facilitate new SPECT system designs by exploiting both enhanced element characteristics and advantageous geometries to achieve high sensitivity as well as a good spatial and energy resolution. In this regard, the first work of this Topic by Besse and Bailly is a case report article describing the use of a new commercial SPECT/CT system equipped with state-of-the-art CZT detectors featuring a ring shaped geometry that enable faster acquisition with better energy resolution. The authors illustrate the use of the StarGuide™ 3D-ring CZT SPECT/CT system (GE Healthcare) in lymphoscintigraphic investigations of lymphoedema. This paper shows a complete SPECT/CT acquisition of the pelvis and limbs with a time reduction of more than 50% compared to a traditional dual-head camera.

The collimator is the component that most influences the image resolution and sensitivity trade-off in a gamma camera. It is an essential component in SPECT to determine the direction of the incident photons, yet posing an upper limit of photon detection. Therefore, novel collimator designs are still actively being pursued to enhance the performance of a SPECT system. In this regard, Wang R. et al. propose a novel cardiac SPECT system without a conventional lead collimator, exploiting with the use of mosaic interval scintillators as collimation to significantly increase sensitivity and reduce scanning time without compromising image resolution. Meanwhile, multi-pinhole collimator is an existing SPECT collimation to improve the sensitivity-resolution trade-off in SPECT particularly for a relatively small field-of-view, e.g., small animals, clinical brain and heart imaging. , In this context, Huang and Mok evaluate the performance of MPH collimator for brain SPECT, exploring its designs along with different number of projection views. They conclude that more number of pinholes are needed for less number of projection views and more complex activity distributions. MPH collimators achieve better spatial resolution and angular sampling than conventional low energy high-resolution (LEHR) and single pinholes in general.

The use of AI is a promising and emerging field that aims to improve the image quality and quantitative accuracy in general medical imaging. The recent advances in machine learning and deep learning techniques are also applied to SPECT, while denoising and attenuation correct are two main applications. In their contribution to this Research Topic, Du et al. compare different deep-learning-based methods of attenuation correction (AC) for ^99m^Tc-TRODAT-1 brain SPECT, a technique for imaging dopamine transporters in patients with Parkinson's disease. Using a 3D conditional generative adversarial network (cGAN), the authors estimated attenuation maps and attenuation-corrected SPECT images by applying different deep learning training strategies. They conclude that deep learning-based AC methods are feasible and robust for DAT SPECT and can improve quantification of dopamine transporter uptake, and indirectly estimating attenuation maps is superior to direct AC by deep learning. Meanwhile, Sun et al. propose a 3D attention-guided generative adversarial network (AttGAN) for denoising fast myocardial perfusion (MP) SPECT images. This novel method uses an attention mechanism to learn the relationships between different regions of the image and improve the quality of the denoised images. The authors compare their method with two other methods using convolutional neural networks (CNN). The results show that for MP-SPECT, AttGAN-based denoising is superior to conventional CNN-based networks.

One of the main advantages of nuclear medicine imaging is the ability to quantify the amount and distribution of a radiotracer in the body. Recently, SPECT has progressed from using relative and semiquantitative measures to using absolute measures of activity concentration, and even further to using normalized uptake with the standardized uptake value (SUV) (8). Within this context, Kaur et al. evaluate the use of quantitative SPECT/CT (Q-SPECT/CT) with ^99m^Tc sulfur colloid to assess disease severity, patient outcomes and therapeutic response to granulocyte colony-stimulating factor treatment in patients with decompensated cirrhosis (DC). The study by Lin et al. reports that whole-body ^99m^Tc methyl-diphosphonate (MDP) bone scans can be used to measure the SUV of bone lesions in patients with lung adenocarcinoma. The paper identifies the maximum SUV as an index that can help identifying bone metastases from benign bone lesions in lung adenocarcinoma patients, especially for those with negative CT findings. The article by Wang X-H et al. show the prognostic value of gated MP SPECT in patients with non-obstructed coronary arteries (INOCA), thus enabling the identification of high-risk groups. The study shows that the long-term predictive efficacy of these data exceeded that of coronary angiography (CAG) data, i.e., the gold standard for detecting obstructive coronary. Consequently, MP SPECT is an accurate tool for predicting the risk of a major adverse cardiovascular event in INOCA.

Radiopharmaceutical therapy has always been a major component in nuclear medicine development. The recent success of radioligand and radioreceptor-based therapies shield a new light for SPECT development, as many theranostic agents also emit gamma photons. SPECT enables voxel-based dosimetry with high accuracy and reproducibility, which is essential for optimizing the efficacy and safety of radiopharmaceutical therapy, as well as predicting and evaluating treatment outcome. However, SPECT-based dosimetry usually requires sequential SPECT scanning with more than 3 imaging time points, making it a clinically challenging practice for most institutions and patients. In their paper, Chen et al. report the use of based on SPECT images at two imaging time points, to simplify dosimetry protocol in a radioligand therapy with ^177^Lu prostate-specific-membrane-antigen. Their results show < 10% dosimetric error as compared to full 4 time points study in both tumors and kidneys.

With continuous advances in instrumentation and computing techniques that improve its diagnostic, quantitation and dosimetric capabilities, SPECT is still a key tool in nuclear medicine. With a wide spectrum of applications clinically and preclinically, SPECT will still be at the forefront of theranostic medicine for decades to come.

## Author contributions

RM: Writing—original draft, Writing—review & editing. GM: Writing—review & editing.
